# Maternal care for preterm infants in the context of the COVID-19 pandemic: a qualitative systematic review

**DOI:** 10.1590/0102-311XEN134924

**Published:** 2025-04-11

**Authors:** Eleonora Pereira Melo, Esther Ferreira dos Santos Mendes, Rita de Cássia Rebouças Rodrigues, Denise Lima Nogueira, Tayná Albuquerque Tabosa, Marcia C. Castro, Márcia Maria Tavares Machado

**Affiliations:** 1 Programa de Pós-graduação em Saúde Pública, Universidade Federal do Ceará, Fortaleza, Brasil.; 2 Faculdade de Medicina, Universidade Federal do Ceará, Fortaleza, Brasil.; 3 Faculdade Luciano Feijão, Sobral, Brasil.; 4 Department of Global Health and Population, Havard T.H. Chan School of Public Health, Boston, U.S.A.; 5 Departamento de Saúde Coletiva, Universidade Federal do Ceará, Fortaleza, Brasil.

**Keywords:** Premature Infant, Mothers, Maternal Behavior, Mother-Child Relations, COVID-19, Recém-Nascido Prematuro, Mães, Comportamento Materno, Relações Mãe-Filho, COVID-19, Recién Nacido Prematuro, Madres, Conducta Materna, Relaciones Madre-Hijo, COVID-19

## Abstract

Preterm birth and hospitalization of the newborn are potentially traumatic events for mothers and children. The COVID-19 pandemic, along with its social impacts and additional concerns, has exacerbated maternal distress and anxiety, adversely impacting the development of premature babies. This study conducted a qualitative systematic review to understand maternal care for preterm infants during the COVID-19 pandemic. Following the Joanna Briggs Institute methodology and PRISMA guidelines, the databases CINAHL, Embase, PsycINFO, Scopus, Web of Science, and the portals Virtual Health Library and CAPES Periodicals were consulted. From the 1,449 identified publications, 14 articles were included in the review, resulting in 172 primary findings. The findings were grouped using the meta-aggregative approach, with confidence assessed via the ConQual approach, resulting in four meta-aggregated findings: (1) the impact of the pandemic on health services for preterm infants; (2) the impact of the pandemic and prematurity on maternal mental health; (3) challenges to the maternal care of preterm infants imposed by COVID-19; and (4) maternal coping strategies during the pandemic. The review revealed that the pandemic and associated sanitary measures negatively impacted maternal proximity to hospitalized premature infants, reduced the support networks, worsened financial situations, and increased mothers’ emotional burden. Public policies are recommended to provide support to mothers, offer resources to deal with adverse experiences, and promote parental skills in caring for preterm infants.

## Abstract (pt)

O nascimento prematuro e a internação do recém-nascido são eventos potencialmente traumáticos para mães e filhos. A pandemia de COVID-19, juntamente com seus impactos sociais e preocupações adicionais, exacerbou a angústia e a ansiedade materna, impactando o desenvolvimento de bebês prematuros. Este estudo realizou uma revisão sistemática qualitativa para compreender o cuidado materno de bebês prematuros durante a pandemia de COVID-19. Seguindo a metodologia do Instituto Joanna Briggs e a declaração PRISMA, foram consultadas as bases de dados CINAHL, Embase, PsycINFO, Scopus, Web of Science e os portais Biblioteca Virtual em Saúde e Periódicos CAPES. Das 1.449 publicações identificadas, 14 artigos foram incluídos na revisão, resultando em 172 achados primários. Os estudos foram agrupados usando uma abordagem de meta-agregação, com confiança avaliada através da abordagem ConQual, resultando em quatro achados meta-agregados: (1) o impacto da pandemia nos serviços de saúde para bebês prematuros; (2) o impacto da pandemia e da prematuridade na saúde mental materna; (3) desafios ao cuidado materno de prematuros impostos pela pandemia de COVID-19; e (4) estratégias de enfrentamento materno durante a pandemia. A revisão revelou que a pandemia e as medidas sanitárias associadas impactaram negativamente a proximidade materna com os bebês prematuros internados, assim como redes de apoio, situação financeira e carga emocional das mães. Recomenda-se a criação de políticas públicas de apoio às mães, assim como oferecer recursos para lidar com experiências adversas e promoção de habilidades parentais no cuidado de prematuros.

## Abstract (es)

El nacimiento prematuro y la hospitalización del recién nacido son eventos potencialmente traumáticos para las madres y los niños. La pandemia del COVID-19 y sus impactos sociales y preocupaciones adicionales han intensificado la angustia y ansiedad materna afectando el desarrollo de los bebés prematuros. Este estudio realizó una revisión sistemática cualitativa para comprender el cuidado materno de los bebés prematuros durante la pandemia del COVID-19. Con base en la metodología del Instituto Joanna Briggs y en la declaración PRISMA, se consultaron las bases de datos CINAHL, Embase, PsycINFO, Scopus, Web of Science y los portales Biblioteca Virtual de Salud y Periódicos CAPES. De las 1.449 publicaciones encontradas en la revisión, se incluyeron 14 artículos, lo que resultó en 172 hallazgos primarios. Los estudios se agruparon utilizando un enfoque de metaagregación, con la confianza evaluada por el enfoque ConQual, lo que resultó en cuatro hallazgos metaagregados: (1) el impacto de la pandemia en los servicios de salud para bebés prematuros; (2) el impacto de la pandemia y la prematuridad en la salud mental materna; (3) desafíos para el cuidado materno de los bebés prematuros impuestos por la COVID-19; y (4) estrategias de afrontamiento materno durante la pandemia. La revisión reveló que la pandemia y las medidas sanitarias asociadas afectaron negativamente la proximidad materna a los bebés prematuros hospitalizados, así como las redes de apoyo a las madres, su situación financiera y la carga emocional de ellas. Se recomiendan crear políticas públicas de apoyo a las madres, así como proporcionar recursos para hacer frente a las experiencias adversas y promover las habilidades parentales en el cuidado de los bebés prematuros.

## Introduction

Neonatal prematurity, defined as birth before 37 weeks of gestation ^1^, is challenging due to the immaturity of the organs and systems of newborns, which renders them more susceptible to infections and complications ^2^ ^,^ ^3^. Intensive care in neonatal units often serves to support the baby’s development and to prevent the occurrence of potential complications ^4^.

The incidence of preterm births has not decreased in recent years. It was estimated that 13.4 million babies will be born preterm in 2020, representing a global prevalence of 9.9% ^1^. This scenario continues to pose a major public health challenge, resulting in significant costs to health systems and significant psychological, social, and financial consequences for families.

The consequences of premature birth, such as the hospitalization of the newborn and the period of separation between family and baby, are potentially traumatic events that can affect the quality of life of mothers and children ^5^. Studies ^6^ ^,^ ^7^ indicate a higher incidence of depression in mothers of premature babies compared to mothers of full-term babies, with negative effects that can persist for months after hospital discharge. This increases the susceptibility of these mothers to illnesses and psychiatric hospitalizations.

The psychological distress caused by the separation of mothers and babies during hospitalization has a detrimental impact on the relationship between mother and child and the care provided. This distress frequently results in women questioning their capacity to fulfill the role of mother, which in turn affects the quality of the mother-baby bond ^8^. The quality of the initial interaction between mother and baby is crucial for the child’s affective and socio-cognitive development, and the first few months of life are fundamental for the evolution of this bond ^9^. A healthy attachment positively impacts both child development and the construction of adequate motherhood, in which the mother takes ownership of her role, understands her child’s needs, and provides them with basic care ^10^.

Furthermore, mothers and families of these preterm infants encounter considerable challenges, such as managing specific aspects of care, including feeding, infection prevention, medication administration, and monitoring growth and neuromaturation, often within contexts characterized by limited resources and inequalities in access to professional support ^1^ ^,^ ^4^. They also contend with the emotional consequences of prematurity, and the transition from hospital to home care, which requires monitoring for delays in motor, cognitive, and behavioral development and providing appropriate sensory stimulation ^6^ ^,^ ^8^.

The onset of the COVID-19 pandemic has resulted in significant psychological distress among pregnant and postpartum women, primarily attributable to social isolation, the threat to their and their infant’s lives, and the concern about not receiving the necessary medical support ^11^ ^,^ ^12^. Additionally, the social inequality that has been exacerbated during this period, in part due to the loss of jobs, has further compounded the challenges faced by this population ^11^ ^,^ ^12^ ^,^ ^13^. Researches ^11^ ^,^ ^14^ indicate that during the pandemic, anxiety, depression, and fear were the frequently reported feelings in this group. Furthermore, the arrival of a new child is a challenge for the family unit, particularly for mothers of preterm babies ^15^.

The adverse social impacts and additional stressors associated with the pandemic can exacerbate maternal distress and anxiety, which in turn can negatively impact the healthy development of premature infants ^16^ ^,^ ^17^. Although there is a substantial body of clinical knowledge regarding the impact of COVID-19 on mothers and newborns, there is a paucity of research specifically investigating the maternal care of preterm children in the context of the pandemic. This highlights the necessity for further longitudinal studies to explore this subject in greater depth. A preliminary search was conducted in the International Prospective Register of Systematic Reviews (PROSPERO), the Cochrane Database of Systematic Reviews, the Joanna Briggs Institute (JBI) Evidence Synthesis, and the Virtual Health Library (VHL) portal, with no current or ongoing systematic review on the topic identified.

Knowledge synthesis studies of qualitative evidence can reveal specific aspects of maternal care in this context, guiding strategies and assisting in public policies to minimize the negative impacts on this vulnerable population. They also provide valuable insights and knowledge for implementing interventions that promote the health and well-being of these families. Therefore, this study aims to conduct a qualitative systematic review to understand maternal care for preterm infants in the context of the COVID-19 pandemic.

## Methodology

This systematic literature review, which was based on qualitative studies, employed a meta-aggregative approach to show a comprehensive understanding of maternal care, focusing on the experiences of the relationship between mother and preterm child. Meta-aggregation, sensitive to nature and traditions of qualitative research, aggregates the findings of primary studies into a combined whole without reinterpretation ^18^. Moreover, it enables the formulation of general recommendations useful for guiding health professionals and policymakers.

A review protocol was drawn up a priori according to the JBI methodology for systematic review and synthesis of qualitative data ^18^, in addition to following the guidelines of the *Preferred Reporting Items for Systematic Review and Meta-Analysis* (PRISMA) ^19^. The review protocol was registered with PROSPERO (registration number CRD42023435717).

The review question was constructed using the PICO strategy ^20^, with the population (P) being “premature child”, the phenomenon of interest (I) “maternal care”, and the context (Co) being the “COVID-19 pandemic”. For this review, a “premature child” is defined as one born before 37 weeks of gestational age ^1^. The term “maternal care” encompasses a broad range of practices, both physical and psychosocial, performed by mothers to promote the well-being and healthy development of their children ^21^.

Thus, the question posed for this review was: How has maternal care for premature infants in the context of the COVID-19 pandemic been addressed in qualitative research? As a secondary question, we have: What are the maternal experiences with premature children in the context of the COVID-19 pandemic?

The search strategy aimed to identify peer-reviewed published studies and was conducted in three stages. First, an initial search limited to the VHL portal was conducted to identify articles addressing this topic. The words in the titles and abstracts of the articles and the index terms that describe them were used to devise a preliminary search strategy. Subsequently, professionals with expertise from the library of the Health Sciences Center of the Federal University of Ceará were consulted to evaluate the initial strategy. Based on their input, a complete search strategy was formulated.

The searched databases were: CINAHL (EBSCOhost), Embase, PsycInfo, Scopus, Web of Science, VHL portal, and CAPES Periodicals portal. All the databases were accessed via the Federated Academic Community (CAFe) of the Capes Periodicals portal. The search strategy, which included all the descriptors identified in the Health Sciences Descriptors (DeCS, acronym in Portuguese) directory and similar terms, was adapted for each database and/or information portal with their corresponding controlled vocabularies (CINAHL Titles, Emtree Terms, APA Thesaurus, and MeSH). Box 1 shows the complete search strategies.

**Box 1:** Search strategies.

DATABASES	SEARCH STRATEGIES
CINAHL	#1 (“Infant, Premature” OR “Childbirth, Premature”) AND Mothers AND (COVID-19 OR “COVID-19 Pandemic”)
	#2 (“Infant, Premature” OR “Childbirth, Premature”) AND (“Maternal Behavior” OR “Maternal-Child Nursing” OR “Maternal-Child Care”) AND (COVID-19 OR “COVID-19 Pandemic”)
	#3 (“Mother-Child Relations”) AND (COVID-19 OR “COVID-19 Pandemic”)
	#4 (“Intensive Care Units, Neonatal” OR “Intensive Care, Neonatal”) AND Mothers AND (COVID-19 OR “COVID-19 Pandemic”)
Embase	#1 (prematurity OR “neonate, premature”) AND (mother OR motherhood OR mothering) AND (“coronavirus disease 2019” OR pandemic)
	#2 (prematurity OR “neonate, premature”) AND (“maternal behavior” OR “maternal caregiving”) AND (“coronavirus disease 2019” OR pandemic)
	#3 (“mother child relation” OR “mother child interaction”) AND (“coronavirus disease 2019” OR pandemic)
	#4 (“neonatal intensive care unit” OR “neonatal ICU”) AND (mother OR motherhood OR mothering) AND (“coronavirus disease 2019” OR pandemic)
PsycINFO	#1 “Premature Birth” AND Mothers AND (COVID-19 OR Pandemics)
	#2 “Premature Birth” AND (“Parental Attitudes (OR “Parental Role”) AND (COVID-19 OR Pandemics)
	#3 (“Mother Child Relations” OR “Maternal Behavior”) AND (COVID-19 OR Pandemics)
	#4 “Neonatal Intensive Care” AND Mothers AND (COVID-19 OR Pandemics)
Scopus and Web of Science	#1 (“Infant, Premature” OR “Infant, Preterm”) AND Mothers AND (COVID-19 OR pandemics)
	#2 (“Infant, Premature” OR “Infant, Preterm”) AND (“Maternal Behavior” OR “Maternal Care Patterns”) AND (COVID-19 OR pandemics)
	#3 “Mother-Child Relations” AND (COVID-19 OR pandemics)
	#4 “Intensive Care Units, Neonatal” AND Mothers AND (COVID-19 OR pandemics)
VHL and CAPES Periodicals portals	#1 (“Recém-Nascido Prematuro” OR Prematuro OR Prematuros OR “Bebê Prematuro” OR “Infant, Premature” OR “Infant, Preterm” OR “Recien Nacido Prematuro” OR “Bebé Prematuro”) AND (Mães OR Mothers OR Madres) AND (COVID-19 OR pandemias OR pandemics)
	#2 (“Recém-Nascido Prematuro” OR Prematuro OR Prematuros OR “Bebê Prematuro” OR “Infant, Premature” OR “Infant, Preterm” OR “Recien Nacido Prematuro” OR “Bebé Prematuro”) AND (“Padrões de Cuidado Materno” OR “Comportamento Materno” OR “Maternal Behavior” OR “Maternal Care Patterns” OR “Conducta Materna” OR “patrones de cuidados maternos”) AND (COVID-19 OR pandemias OR pandemics)
	#3 (“Relações Mãe-Filho” OR “Mother-Child Relations” OR “Relaciones Madre-Hijo”) AND (COVID-19 OR pandemias OR pandemics)
	#4 (“Unidades de Terapia Intensiva Neonatal” OR “Intensive Care Units, Neonatal” OR “Unidades de Cuidado Intensivo Neonatal”) AND (Mães OR Mothers OR Madres) AND (COVID-19 OR pandemias OR pandemics)

The review exclusively considered research employing qualitative methodological designs. The studies should investigate the perceptions, views, experiences, attitudes, and beliefs of mothers, fathers, and/or health professionals about maternal care, with a particular focus on the experiences of mother-preterm child relationships during the COVID-19 pandemic. The review included articles published from 2020 to October 2023 in Portuguese, English, and Spanish. The articles were available in full text and with unrestricted access. Articles employing quantitative or mixed methodologies, review studies, doctoral theses, master’s dissertations, specialization or undergraduate monographs, opinion articles, reviews, editor’s letters, and commentaries were excluded.

After the search, all the identified references were exported to Rayyan (https://www.rayyan.ai/), a web application designed to assist with systematic reviews and meta-analyses ^22^, and duplicate publications were removed. Two independent and blinded reviewers then examined the titles and abstracts to determine whether they met the inclusion and exclusion criteria for the review. All reasons for exclusion were recorded, and the screening results of the two reviewers were cross-checked. Discrepancies were resolved by a discussion with a third reviewer with experience in the subject matter. Subsequently, all articles that met or could potentially meet the inclusion criteria were retrieved and their details were imported into Mendeley reference manager (https://www.mendeley.com). The full texts of the selected articles were retrieved for reading and subject to a detailed analysis in relation to the inclusion and exclusion criteria. The entire selection process was elucidated in a PRISMA flowchart.

The included studies were critically appraised for methodological quality using the *JBI Critical Appraisal Checklist for Qualitative Research* ^23^. Two independent reviewers used the *JBI Qualitative Assessment and Review Instrument* (JBI QARI) ^18^ to extract qualitative data from the included articles. According to this instrument, information extracted from each study included details about the phenomenon investigated, the characteristics of the participants, the methods for data collection and analysis, the geographical location, the context or the culture, and the main findings relevant to the review question.

Subsequently, the extraction with the JBI QARI involved retrieving the findings and illustrations from each study, which were extracted and assigned to a level of credibility: unequivocal, credible, or not compatible. The findings, which are the analytical interpretations of the authors of the reviewed studies, are extracted verbatim and accompanied by illustrations, which are the statements of the subjects who took part in these studies. Findings are considered unequivocal when they are accompanied by an illustration that is beyond reasonable doubt. Credible findings are accompanied by an illustration that does not have a clear association with the findings in question, making them open to challenge. A finding is non-compatible when it is not supported by an illustration ^18^.

The findings and the respective illustrations were grouped using the qualitative data analysis software MaxQDA (https://www.maxqda.com) with the meta-aggregation approach ^18^. This entailed the aggregation of findings that were deemed similar into categories, accompanied by an explanatory statement that conveyed the inclusive meaning of the group of findings. These categories were then synthesized into a comprehensive set of synthesized findings that can be used for evidence-based practice. Only unequivocal and credible findings were included in the synthesis.

The synthesized results were evaluated using the ConQual approach ^24^ to ascertain the trustworthiness and credibility of the qualitative research synthesis. The reliability of the findings was assessed via the application of five questions measuring the congruence with the *JBI Critical Appraisal Checklist for Qualitative Research* ^18^. The credibility was determined by assessing the congruence between the authors’ interpretations and the available data. ConQual then categorized and assigned a confidence level to the qualitatively synthesized results (high, moderate, low, or very low), based on a comprehensive consideration of the reliability and credibility assessment.

## Results

### Description of the sample

**Figure 1:**
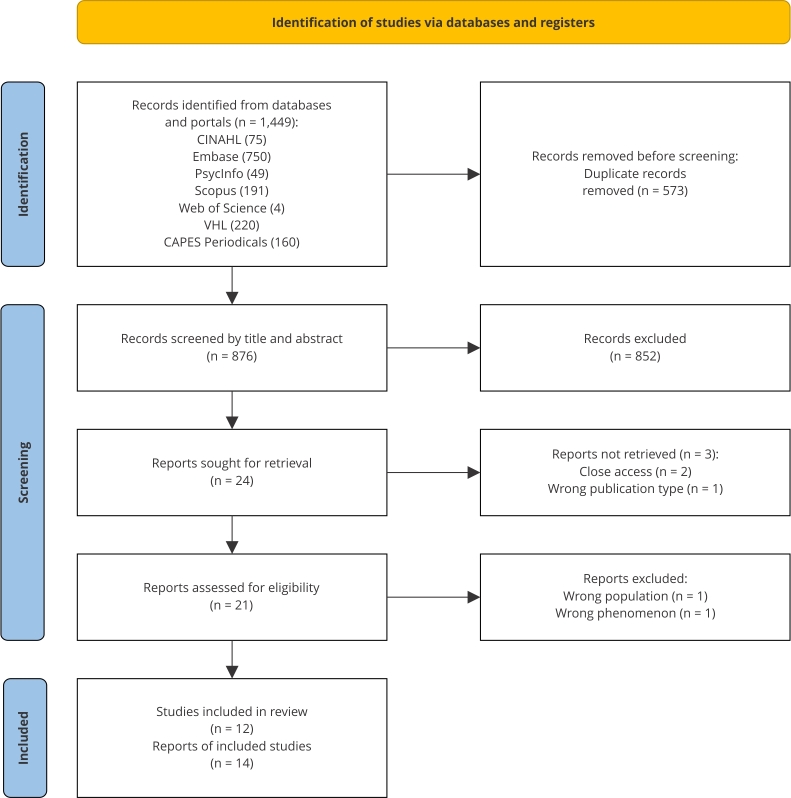
*Preferred Reporting Items for Systematic Review and Meta-Analysis* (PRISMA) flowchart with the entire selection process.

Of 1,449 publications, 14 articles were included according to the flowchart (Figure 1). Box 2 summarizes the main characteristics of the articles. The analysis of the characteristics of the articles revealed that 5 (35.71%) were published in 2021, 6 (42.85%) in 2022, and 3 (21.42%) in 2023. Two (14.28%) articles used open-ended online questionnaires for data collection, while 12 (85.71%) used telephone interviews or video calls. Of the studies, 6 (42.85%) took place in Brazil, 3 (21.42%) in the United States, and 1 (7.14%) study each in Poland, the United Kingdom, Norway, Ethiopia, and Sweden. All articles (100%) were published in English, 6 (42.85%) in Brazilian journals, and 8 (57.14%) in international journals. Ten (71.42%) articles focused on parents’ experiences with preterm infants admitted to a neonatal unit during the COVID-19 pandemic, 3 (21.42%) on outpatient follow-up, and 1 (7.14%) study included both contexts. Five (35.71%) publications focused exclusively on maternal perceptions, 8 (57.14%) also considered paternal perspectives, and 1 (7.14%) included healthcare professionals’ perceptions.

**Box 2:** Characteristics of the included studies according to the *JBI Qualitative Assessment and Review Instrument* (JBI QARI).

STUDY (YEAR)/COUNTRY	METHODS FOR DATA COLLECTION AND ANALYSIS	PHENOMENON OF INTEREST	SETTING/CONTEXT/CULTURE	PARTICIPANT CHARACTERISTICS AND SAMPLE SIZE	DESCRIPTION OF THE MAIN RESULTS
Kynø et al. ^25^ (2021)/Norway	Semi-structured interviews were recorded and transcribed. The information was subjected to inductive thematic analysis	The experience of parents who had children hospitalized in the NICU during the visitation ban due to the COVID-19 pandemic	Department of Neonatal Intensive Care, Department of Childbirth and Department of Cardiothoracic Surgery in Norway. The newborns were hospitalized for at least 14 days during the most comprehensive restriction period of the pandemic and were discharged from the NICU at the time of the invitation to participate in the research	9 mothers and 4 fathers with premature, very premature, or full-term babies. The average length of stay for newborns in the NICU was 59 days	Reports of delayed bonding or a different connection with the child; difficulty in developing the experience of being a family without being together, supporting each other; being present with the child during visiting restrictions generated regret and frustration, as well as altering the family dynamic beyond hospitalization in the NICU; presence of feelings of loneliness in mothers, sometimes related to the absence of fathers to share experiences
Mengesha et al. ^26^ (2022)/Ethiopia	Semi-structured in-depth interviews. The interviews were recorded, transcribed, and translated into English. The transcript was checked by reading and listening to the interviews repeatedly. The information collected was subjected to thematic analysis and Open Code software was used to process the data	The experiences of parents of premature babies admitted to the NICU	Ethiopian hospital established in 1961. It currently has seven wards, including a neonatal ICU. This unit was established in 2015 and has the support of 6 doctors and 31 nurses who look after 83 beds, including: the Kangaroo Mother Method with 8 beds, the maternal side with 33 beds, the term side with 14 cribs, the preterm room with 20 cribs and the septic room with eight beds. The NICU ward was full and there was no space for family members and visitors to rest, there were no waiting rooms or chairs	18 parents were interviewed, 9 mothers and 9 fathers. All were Orthodox Christians. Most were married and farmers, which affected the length of time these parents spent in hospital due to the harvest period	Accompanying the long hospitalization of hospitalized babies led to financial difficulties; most parents felt anxious, stressed, worried, hopeless, and confused, which has a great influence on the parental experience in the NICU; parental involvement has a great contribution to the improvement of premature babies; parents faced limited access to medication, water shortages, inadequate spaces for rest and restrictions on visitation, resulting in dissatisfaction and financial difficulties; constant worry about their child’s health condition; the context of the COVID-19 pandemic and the seasonal situation (agricultural season) influenced parents’ stay in hospital
Marino et al. ^27^ (2022)/United Kingom	Participants were recruited via snowball sampling via the social networks of the charity Baby Life Support System (Bliss) and the Neonatal Unit at Princess Anne Hospital, Southampton. An online questionnaire was administered with open-ended questions on the following topics: experience, information, decision-making process and support needs. The information was subjected to thematic content analysis	The experience, information and support needs and decision-making of parents with a premature or sick newborn during the COVID-19 pandemic	United Kingdom residents during social isolation, when many women gave birth alone, except for the presence of health professionals. In addition to reduced parental access, especially with babies who were born prematurely, only one parent was allowed to visit at a time and no additional visitors	107 parents took part, 103 mothers and four fathers. The average age was 29.5 years. 50% of preterm babies were born before 33 weeks of gestational age	Many parents reported significant psychological and emotional impact due to the restrictions in the birth experience and afterwards and in their neonatal intensive care journey; feelings of loneliness and impact on the maternal bond with their child, whether due to the face covered by the mask or the concern about the child’s ability to distinguish the parents’ voice, touch and smell among the multidisciplinary team
Spence et al. ^28^ (2023)/United States	Semi-structured interviews conducted individually via videoconference. The interviews were audio-recorded, transcribed and subjected to collaborative qualitative analysis	The experiences of parents of premature babies who were hospitalized in the NICU and their transition home	NICU of a Virginia State hospital	12 mothers and 1 father of very premature babies (< 29 weeks) who had already been discharged from the NICU 8 weeks previously	The multiple challenges faced were highlighted, from separation from the baby in the first months of life to logistical and emotional difficulties. The sudden transition to home, the anxiety surrounding discharge and the loss of support from the nursing team were pointed out as generating concern and dissatisfaction. During the COVID-19 pandemic, there was a reduction in the support network and difficulty communicating with health professionals. The results highlight the importance of providing mental health care to parents, as well as creative approaches to offering support, including frequent communication with health professionals, participation in care activities and the creation of support networks between families
Vance et al. ^29^ (2021)/United States	Participants were recruited via social networks. The answers were transferred to NVivo 11 software and subjected to thematic content analysis	The experience of parents with children admitted to the NICU during the COVID-19 pandemic	Residents of 38 states in the United States who had children admitted to the NICU during the first wave of COVID-19	169 participants, 164 of them mothers, with an average age of 31	Parents’ experiences of having children hospitalized in the NICU during the COVID-19 pandemic have been emotionally draining and isolating; policies restricting parental presence have created ruptures in the family unit and limited basic care; communication with NICU professionals has been related to intensifying and alleviating the emotional suffering of different mothers and fathers
Lindgren et al. ^30^ (2023)/Sweden	The semi-structured interview was conducted by videoconference, telephone, or in person at the interviewee’s home. The interview was recorded and transcribed for subsequent qualitative content analysis using an inductive approach. Microsoft Excel was used to manage the information	Exploring the experience of parents who have been separated from their children due to COVID-19	2 NICUs in university hospitals in central Sweden	There were 11 parents with 7 newborns, 7 mothers and 4 fathers who were separated from their children after birth due to a positive COVID-19 test. The average gestational age of newborns was 30 weeks, with an average birth weight of 1,572g. The mean age of the parents was 30 years. Five were married and 6 were cohabiting. Regarding schooling, 7 had higher education	Parents being separated from their newborn was related to feelings of abandonment, loneliness and uncertainty about the unsafe situation. Video calls with their children were seen as essential. Hope and faith were coping strategies used by the parents to help in this difficult situation. Feelings of grief at the loss of first contact with their child were reported. Feelings of fear that their child would forget or not recognize their parents. Difficulty in recognizing themselves as parents during separation, as well as a loss of parental self-confidence. There was a common report of a desire to reunite with their child, to be close to them, to make skin-to-skin contact to help their relationship and bond. The separation affected the newborn’s relationship with their parents in the long term, with stress reported up to a year after discharge
Silva et al. ^31^ (2021)/Brazil	Online interviews using instant messaging application, collected via recorded messages and audios, which were transcribed in full for later analysis via the interpretation of meanings	Elements related to monitoring the health of children with a history of prematurity during the COVID-19 pandemic	Children’s Nutrition Center, aimed at users of public health services, in the city of Foz do Iguaçu, Paraná State, which belongs to the triple border with Ciudad del Este (Paraguay) and Puerto Iguazú (Argentina)	12 mothers with 14 children (2 were twins). The children were born prematurely with a gestational age of 30 to 35 weeks and a length of hospital stay after birth of 5 to 58 days. The children’s ages during the study ranged from 2 years and 7 months to 2 years and 10 months. All the mothers were over 18 years old	The COVID-19 pandemic and the child’s health: beginning to adopt hygienic habits in order to reduce the risk of contamination; concern about prematurity being an aspect that can weaken health at this time of the pandemic and about family life being a risk factor; concern about poor medical care, due to the suspension of the service; social distancing and the repercussions on the child’s development: social isolation was a cause for concern about the development of the child, who showed irritation, tiredness and requests for more presence and interaction; difficulty in carrying out daily activities with the child at home; institution of a routine to cope with difficulties; there were feelings of uncertainty about the maternal role; challenges in following up the health of the premature child: presence of family problems, difficulties in following up the support of health professionals; maternal understandings and challenges in caring for mothers when faced with the presence of flu-like symptoms in the child
Reichert et al. ^32^ (2022)/Brazil	Semi-structured interviews conducted individually by telephone. The interviews were transcribed and subjected to the inductive thematic analysis technique	The experiences of mothers of infants who were born prematurely during the COVID-19 pandemic	Preterm birth follow-up clinic at a public maternity hospital in a municipality in the state of Paraíba	21 mothers, aged between 18 and 38. Nine had 1 child, 7 had 2 children and 5 had 3 or more children. In terms of years of schooling, 14 women had between 5 and 10 years, and 7 had more than 10 years of schooling	Maternal perception of COVID-19: overload of information in the media about the disease, prevention methods, etc. Different opinions on social isolation, sometimes negative, sometimes positive; experiences of mothers of premature infants: increased vulnerability of mothers and families; socioeconomic situation due to unemployment; restriction of meetings with family members; little support from the care network, generating changes in the psychological condition; in addition to maternal stress in mothers with other children, as they had to pay attention to other children who were now idle at home
Aftyka et al. ^33^ (2023)/Poland	Individual telephone interviews. The interviews were recorded and transcribed. A thematic analysis using inductive coding was carried out using NVivo qualitative data analysis software	Opinions and emotional reactions of parents who received photographs or videos of their children in a NICU	NICU at the Children’s University Hospital in Lublin. The unit was closed to parents and other visiting relatives due to the COVID-19 pandemic	17 parents of hospitalized newborns, 4 male (fathers) and 13 female (mothers). The babies were in different periods and courses of treatment, with a variety of health problems and prognoses, among them premature newborns	Usefulness of the intervention: photos and videos were considered important tools by parents to accompany their babies at times when they can’t be present in the neonatal unit, complementing the team’s support; parents’ emotional reaction: negative with the first photos, evolving into positive emotions over time. Anxiety and depression were common during their child’s stay in the NICU; potential ways to improve the intervention: mothers showed interest in visits and frequent communication with the medical team; use of a webcam and family beds in the NICU were pointed out as promising interventions
Rocha & Dittz ^34^ (2021)/Brazil	Semi-structured interview conducted in person. The interview was recorded and transcribed for later thematic content analysis	The repercussions of social isolation on the daily lives of mothers of babies admitted to the NICU during the COVID-19 pandemic	Comprehensive Health Care Foundation/Sofia Feldman Hospital, in Belo Horizonte (Minas Gerais State), which specializes in maternal and childcare and exclusively serves users of the SUS. In addition to the obstetric beds, the institution has 51 NICU beds and provides conditions for mothers to stay full-time during their newborn’s stay in the NICU, called the Sofia Space	15 mothers who had spent more than 5 days in Sofia Space and had babies in the NICU for more than 7 days. The mothers’ ages ranged from 19 to 37. All had an average of 11 years of schooling	Access to information and knowledge about COVID-19: some mothers said they had some knowledge about the pandemic. In addition to uncertainties about the disease, such as the possibility of a cure and treatment, they also say they adhere to new habits, terms and recommendations; repercussions on the way they live: preventive measures have become part of their daily lives; intensified concern about preventive measures due to their babies being hospitalized in the NICU and also the impact of social restriction and how this has reverberated in the family support provided; being the mother of a baby hospitalized in the NICU during COVID-19: fear of possible contamination of their children, added to the concern because they are already premature, as well as feelings such as impotence, anguish and sadness; fear of contaminating the baby, avoiding exercising care in the NICU, such as touching and holding the baby
Joaquim et al. ^35^ (2022)/Brazil	Demographic questionnaire and semi-structured interview. The interviews were recorded, transcribed in full and subjected to content analysis by 2 researchers	The experience of women in pregnancy or puerperium in the context of the pandemic during care at a referral hospital	A public maternity hospital linked to the SUS, specializing in care for women and children located in Belo Horizonte (Minas Gerais State). The mothers recruited were those being cared for in the assistance programs, pregnant women being monitored in hospital, mothers of newborns hospitalized in NICU and mothers of newborns treated at the Risk Newborn Follow-up Clinic	18 women took part, 10 primiparous and 8 multiparous, aged between 19 and 42. Among the pregnant women, the gestational age ranged from 26 weeks and 4 days to 35 weeks and 3 days. Among the puerperal women, the newborns had gestational ages between 26 weeks and 39 weeks and 3 days	Repercussions on pregnancy and puerperium: there was fear, anxiety and worry among pregnant women, which affected their interactions with their babies and increased their concerns about the health and future of their newborns, especially when they were premature. Social distancing and lack of family support aggravated the emotional situation; repercussions of COVID-19 on practical life: the pandemic’s preventive measures significantly altered mothers’ routines, intensifying the use of masks, hand hygiene and social distancing, also affecting work and social interaction. In addition, some maternal care, such as touching or holding the hospitalized baby, decreased; coping strategies: meditation, various occupations and the use of information technology to preserve mental health during the pandemic, while interaction with other mothers in the hospital provided emotional support and shared experiences
Reichert et al. ^36^ (2021)/Brazil)	Semi-structured interview conducted individually over the phone, which was recorded. The inductive thematic analysis technique was used for analysis	The impact of COVID-19 on the care of premature babies, from the perspective of mothers and health professionals	Preterm birth follow-up clinic at a public maternity hospital in a municipality in the state of Paraíba	14 mothers of infants who were born prematurely and 4 health professionals. The mothers were aged between 23 and 38, of whom 3 had less than 8 years of schooling, and 11 had 8 years or more	Repercussions of the COVID-19 pandemic on care for premature infants: lack of monitoring of premature infants in the health care network. Maternal fear of exposing the child to the disease. Low socioeconomic status; coping strategies for organizing outpatient care for preterm infants during the COVID-19 pandemic: monitoring by telephone; increased hygiene measures; compliance with the standards recommended by health surveillance; spacing appointments further apart and reducing the number of children seen
Rocha et al. ^37^ (2022)/Brazil	Individual interviews were conducted over the phone and guided by a script containing: (1) sociodemographic and obstetric data; (2) guiding questions. The interviews were recorded and transcribed. Descriptive statistics were used to analyze the sociodemographic and reproductive data, using IBM SPSS 22.0. To analyze the information, we used thematic content analysis processed using IRAMUTEQ software, adopting the Descending Hierarchical Classification	Perceptions of mothers of premature newborns admitted to the neonatal unit in the face of the coronavirus pandemic	Neonatal unit of a reference maternity hospital for newborn health care in the state of Ceará, northeastern Brazil	12 mothers of premature babies who were admitted to the neonatal unit of this institution	The perception of these mothers is centered on the difficulties faced during this period, with negative feelings due to the distance between mother and child, since visitation was avoided during the babies’ hospitalization due to COVID-19. Despite this, it was possible to observe the mothers’ hope in the return of face-to-face visits and in meeting their child
Vance et al. ^38^ (2022)/United States	Participants were recruited via social networks. The answers were transferred to NVivo 11 software and subjected to thematic content analysis	Parents’ concerns and reactions to NICU visitation policies related to the COVID-19 pandemic	Residents of 36 states in the United States who had children admitted to the NICU during the first wave of COVID-19	A total of 155 parents, 149 mothers and 6 fathers, with an average age of 30. 72% were married and 75% declared themselves white. Prematurity was the most common reason for admission to the NICU (72%), with an average length of stay of 38 days	Parents’ experiences in the NICU during the COVID-19 pandemic have been emotionally overwhelming, traumatizing and stressful, with widespread feelings of loneliness and isolation; restrictive policies regarding parents’ presence have undermined parents’ essential role as caregivers and resulted in significant losses, including interference in bonding, shared experiences and impaired communication with specialists, exacerbating grief, isolation and anger among parents; and interactions with NICU professionals have intensified or alleviated the emotional distress felt by parents. There were feelings of alienation from the care team, which was attributed to the changes due to COVID-19

ICU: intensive care unit; NICU: neonatal intensive care unit; SUS: Brazilian Unified National Health System.

Regarding the critical appraisal of the articles (Box 3), the methodological quality of the studies was assessed as moderate. Although 6 (42.85%) articles clearly demonstrated alignment between their methodology and philosophical perspective, 13 (92.85%) showed congruence between methodology and research questions or objectives. All publications (100%) maintained coherence between methodology, data collection methods, and the presentation and analysis of data, with 13 (92.85%) also aligning their methodology with the interpretation of results. However, only 5 (35.71%) articles adequately situated the researcher culturally or theoretically, and 2 (14.28%) acknowledged the reciprocal influence between the researcher and the research. Thirteen (92.85%) studies appropriately described their participants and their voices, and all studies (100%) received ethical approval.

**Box 3:** Critical appraisal results to the *JBI Critical Appraisal Checklist for Qualitative Research*.

STUDY	QUESTIONS									
	1	2	3	4	5	6	7	8	9	10
Kynø et al. ^25^ (2021)	Unclear	Yes	Yes	Yes	Yes	Yes	Yes	Yes	Yes	Yes
Mengesha et al. ^26^ (2022)	Yes	Yes	Yes	Yes	Yes	No	No	Yes	Yes	Yes
Marino et al. ^27^ (2022)	Unclear	Yes	Yes	Yes	Yes	No	No	Yes	Yes	Yes
Spence et al. ^28^ (2023)	Yes	Yes	Yes	Yes	Yes	No	Unclear	Yes	Yes	Yes
Vance et al. ^29^ (2021)	Yes	Yes	Yes	Yes	Yes	No	No	Yes	Yes	Yes
Lindgren et al. ^30^ (2023)	Unclear	Yes	Yes	Yes	Yes	No	No	Yes	Yes	Yes
Silva et al. ^31^ (2021)	Yes	Yes	Yes	Yes	No	No	No	Yes	Yes	Yes
Reichert et al. ^32^ (2022)	Unclear	Yes	Yes	Yes	Yes	No	No	Yes	Yes	Yes
Aftyka et al. ^33^ (2023)	Yes	Yes	Yes	Yes	Yes	No	No	Yes	Yes	Yes
Rocha & Dittz ^34^ (2021)	Yes	Yes	Yes	Yes	Yes	Yes	Unclear	Yes	Yes	Yes
Joaquim et al. ^35^ (2022)	Unclear	Yes	Yes	Yes	Yes	Yes	Yes	Yes	Yes	Yes
Reichert et al. ^36^ (2021)	Unclear	Yes	Yes	Yes	Yes	Yes	No	Yes	Yes	Yes
Rocha et al. ^37^ (2022)	Unclear	Yes	Yes	Yes	No	Yes	No	Unclear	Yes	Unclear
Vance et al. ^38^ (2022)	Unclear	Unclear	Yes	Yes	Yes	No	No	Yes	Yes	Yes
%	42.85	92.85	100.00	100.00	85.71	35.71	14.28	92.85	100.00	92.85

Questions: Q1. Is there congruity between the stated philosophical perspective and the research methodology?; Q2. Is there congruity between the research methodology and the research question or objectives?; Q3. Is there congruity between the research methodology and the methods used to collect data?; Q4. Is there congruity between the research methodology and the representation and analysis of data?; Q5. Is there congruity between the research methodology and the interpretation of results?; Q6. Is there a statement locating the researcher culturally or theoretically?; Q7. Is the influence of the researcher on the research, and vice versa, addressed?; Q8. Are participants, and their voices, adequately represented?; Q9. Is the research ethical according to current criteria or, for recent studies, and is there evidence of ethical approval by an appropriate body?; Q10. Do the conclusions drawn in the research report flow from the analysis, or interpretation, of the data?.

### Meta-aggregated findings

A total of 172 primary findings were extracted from the 14 articles reviewed, and aggregated into 21 categories and later synthesized into four meta-aggregated findings: (1) the impact of the pandemic on health services for preterm infants; (2) the impact of the pandemic and prematurity on maternal mental health; (3) challenges to the maternal care of preterm infants imposed by COVID-19; and (4) maternal coping strategies during the pandemic. The results are in a summary of findings (Box 4), which provides a detailed explanation of the development of the ConQual score.

**Box 4:** Summary of metasynthesized findings with ConQual.

SYNTHESIZED FINDINGS	DEPENDABILITY	CREDIBILITY	ConQual SCORE	COMMENTS
1. The impact of the pandemic on health services for preterm infants: the changes imposed by the pandemic on health services have had repercussions on maternal care for premature infants and on monitoring the baby’s health	Downgraded 1 level	Downgraded 1 level	Low	Low dependability: out of 10 primary studies, 3 studies scored positive for 4 to 5 dependability questions; 7 scored 3 dependability questions (they did not locate the researcher culturally or theoretically and did not address the researcher’s influence on the research and vice versa). Credibility: downgraded due to a mix of unequivocal and credible findings (21 unequivocal and 13 credible)
2. The impact of the pandemic and prematurity on maternal mental health: the pandemic has increased the emotional burden on mothers, overriding their existing fears about the premature birth of their baby and their health	Downgraded 1 level	Downgraded 1 level	Low	Low dependability: out of 11 primary studies, 5 studies scored positive for 4 to 5 dependability questions; 6 scored 3 dependability questions (they did not locate the researcher culturally or theoretically and did not address the researcher’s influence on the research and vice versa). Credibility: downgraded due to a mix of unequivocal and credible findings (23 unequivocal and 19 credible)
3. Challenges for maternal care of preterm infants imposed by COVID-19: the COVID-19 pandemic and the health measures to prevent it have imposed extra challenges on mothers, as they have compromised maternal closeness and parental exercise with the preterm child hospitalized in a neonatal unit, reduced the support network, affected the financial situation and increased the emotional and care burden with the baby	Downgraded 1 level	Downgraded 1 level	Low	Low dependability: out of 10 primary studies, 4 studies scored positive for 4 to 5 dependability questions; 6 scored 3 dependability questions (they did not locate the researcher culturally or theoretically and did not address the researcher’s influence on the research and vice versa). Credibility: downgraded due to a mix of unequivocal and credible findings (31 unequivocal and 24 credible)
4. Maternal coping strategies during the pandemic: mothers resorted to various support alternatives, whether for practical support in caring for their children or to strengthen themselves emotionally in the face of the troubled times they were going through; there were changes in routines and habits in caring for premature babies, with adherence to new measures to protect and prevent the disease	Downgraded 1 level	Downgraded 1 level	Low	Low dependability: out of 10 primary studies, 4 studies scored positive for 4 to 5 dependability questions; 6 scored 3 dependability questions (they did not locate the researcher culturally or theoretically and did not address the researcher’s influence on the research and vice versa). Credibility: downgraded due to a mix of unequivocal and credible findings (29 unequivocal and 12 credible)

#### Synthesized finding 1: the impact of the pandemic on health services for preterm infants

The pandemic had a significant impact on health services, resulting in changes in care routines, restrictions on neonatal unit visits, suspension of preterm infant follow-up services, and overburdening health professionals. These changes affected maternal care and health monitoring of preterm infants after discharge. To mitigate the effects of the social distancing measures, online interventions were implemented both in the neonatal units and in the follow-up services. These interventions aimed to maintain proximity, monitor the babies’ development, and provide support and guidance to families in caring for their children.

This finding was synthesized by aggregating four categories derived from 34 primary findings extracted from studies ^25^ ^,^ ^26^ ^,^ ^27^ ^,^ ^28^ ^,^ ^29^ ^,^ ^30^ ^,^ ^31^ ^,^ ^32^ ^,^ ^33^. Twenty-one of these findings were unequivocal and 13 were credible. The four aggregated categories were: (1.1) disorganization and disruption of services in the pandemic, supported by 7 findings (6 unequivocal and 1 credible); (1.2) restriction or suspension of access to neonatal units, supported by 5 findings (2 unequivocal and 3 credible); (1.3) interruption or restriction of healthcare, supported by 11 findings (6 unequivocal and 5 credible); (1.4) incorporation of the use of information and communication technologies in care, supported by 11 findings (7 unequivocal and 4 credible).

### (a) Category 1.1: disorganization and disruption of services in the pandemic

Mothers expressed dissatisfaction and frustration with the services that were provided during delivery and in the neonatal intensive care unit (NICU). They cited delays, a lack of leadership, disorganization, insufficient or mismatched information, and inconsistency in the restrictions applied ^25^ ^,^ ^26^. Many of these mothers perceived that some of the policies regarding access to the NICU were inappropriate, such as seemingly random visiting hours and arbitrary changes in the rules for NICU stay during the pandemic ^27^. The lack of meetings with family members to inform them about the infant’s health status or the communication of the medical report to only one parent was a source of dissatisfaction, as both parents should be involved ^28^. Furthermore, there have been complaints about the inconsistent adherence of NICU professionals to protective measures against COVID-19 ^29^.

### (b) Category 1.2: restriction or suspension of access to neonatal units 

The restrictions on visits to children in the NICU during the pandemic had a significant impact on the duration of mothers’ stays in the neonatal unit and made it challenging for them to access information about their babies when they were unable to be present ^26^ ^,^ ^29^. The restrictions resulted in feelings of isolation and exclusion from the care of their child ^29^. Some mothers proposed that, if they were unable to be with their infants other family members could assume as substitutes, or volunteers could assist the healthcare professionals, as they felt they might not be able to fully meet the newborn’s care and attention needs ^27^ ^,^ ^30^.

### (c) Category 1.3: interruption or restriction of healthcare

The disruption of health services during the pandemic led to heightened maternal concerns about their children’s health ^31^. The mothers needed more physical and emotional support, but the restrictions prevented them from receiving proper attention ^28^. Although the provision of support from health professionals was regarded as crucial during the NICU stay, it frequently proved to be inadequate, with some professionals displaying a lack of commitment ^26^. The deactivation of the kangaroo unit during the period further restricted the opportunity for close contact between mother and baby ^32^. Furthermore, mothers expressed frustration knowing they would not have NICU team assistance after discharge ^28^.

Several premature infant follow-up services were terminated ^31^, while one follow-up clinic reduced the number of children seen and increased the spacing between appointments, focusing only on the most urgent cases ^32^. Similarly, follow-up of premature infants in primary healthcare was reduced or discontinued ^32^. Other health services have altered their care routines to prioritize the management of COVID-19, which may compromise the care of premature children with underlying health conditions ^31^. The reduction in the number of teams has led to an increase in the workload of professionals, with members leaving due to illness or belonging to risk groups, affecting the provision of care ^32^.

### (d) Category 1.4: incorporation of the use of information and communication technologies in care

During the pandemic’s most restrictive period, resources such as telephone or video calls, sending images of the hospitalized newborn, and remote consultations were employed ^30^ ^,^ ^32^ ^,^ ^33^. Although mothers preferred face-to-face contact, they considered receiving images and videos of their hospitalized child useful for monitoring the infant’s health, providing a sense of closeness, and facilitating introductions to other family members ^33^. Nevertheless, some mothers preferred the alternative of seeing their children through a window with subsequent face-to-face communication updates ^33^. The incorporation of telehealth services post-discharge was valuable for monitoring the premature infant’s health ^32^.

#### Synthesized finding 2: impact of the pandemic and prematurity on maternal mental health

The emotional burden on mothers of premature babies has increased significantly because of the COVID-19 pandemic. The emotional distress associated with their child’s prematurity and health was further compounded by the emotional impact of the pandemic, particularly for those with hospitalized infants.

This finding was synthesized by aggregating four categories that derived from 42 primary findings extracted from studies ^25^ ^,^ ^26^ ^,^ ^27^ ^,^ ^28^ ^,^ ^29^ ^,^ ^30^ ^,^ ^31^ ^,^ ^32^ ^,^ ^34^ ^,^ ^35^ ^,^ ^36^ ^,^ ^37^. Twenty-three of the findings were unequivocal and 19 were credible. The four aggregated categories were: (2.1) emotional repercussions of premature birth on mothers, supported by 10 findings (5 unequivocal and 5 credible); (2.2) fear of contamination, supported by 10 findings (5 unequivocal and 5 credible); (2.3) isolation and loneliness, supported by 8 findings (7 unequivocal and 1 credible); (2.4) psychic suffering caused by the pandemic, supported by 14 findings (6 unequivocal and 8 credible).

### (a) Category 2.1: emotional repercussions of premature birth on mothers

The experience of prematurity and the newborn’s NICU hospitalization resulted in traumatic, fearful, and isolating effects that persisted beyond the infant’s discharge ^26^ ^,^ ^28^. Processing medical information about the premature infant was challenging, and the risk of mortality associated with prematurity impeded strong maternal-family bonds, hindering mothers’ preparation to take their children home ^28^. The transition in care responsibility post-discharge heightened maternal concerns as they became the primary caregivers ^28^. Despite being a long-awaited moment viewed with gratitude, the infant’s discharge caused anxiety and distress ^28^.

### (b) Category 2.2: fear of contamination

The fear of COVID-19 contamination was pervasive among mothers of premature infants, particularly those who were admitted to the NICU ^34^. This fear led some mothers to accept the separation imposed in neonatal units, which were perceived as safer environments for their children ^30^ ^,^ ^34^ ^,^ ^35^. Additionally, they were concerned that family members would not adhere to the recommended preventive measures and could transmit COVID-19 to the infant ^31^. Some mothers experienced a conflict between caring for their NICU infants and protecting other children at home ^34^. Furthermore, mothers were reluctant to attend post-discharge follow-up appointments due to concerns regarding exposure to the virus, which may have had adverse effects on the child’s health ^32^.

### (c) Category 2.3: isolation and loneliness

Pregnancy and having a premature birth during the pandemic were described as lonely experiences ^27^. COVID-19 restrictions reduced or even made it impossible to share the pregnancy and birth with other family members ^35^. Similarly, the NICU stay of the infant during the pandemic was described as extremely lonely due to the absence of family members ^27^ ^,^ ^29^. Fathers or other family members had no access to the NICU, so mothers were solely responsible for informing the family about the baby’s health, contributing to feelings of loneliness ^25^. Despite professional support being available, some mothers did not utilize it, while others did not receive the necessary support when they needed it ^28^.

### (d) Category 2.4: psychic suffering caused by the pandemic

Giving birth without seeing or holding the child immediately, and being separated from the family, was described as extremely difficult ^25^ ^,^ ^28^ ^,^ ^29^ ^,^ ^36^. Furthermore, the infant’s hospitalization in the NICU during the pandemic, coupled with restricted contact, was depicted as traumatic ^29^ ^,^ ^30^ ^,^ ^37^. Mothers reported greater distress, uncertainty about the future, and increased worry ^28^ ^,^ ^29^ ^,^ ^35^. Other negative emotions included fear, anguish, sadness, and feelings of helplessness ^30^ ^,^ ^34^. The separation from their hospitalized children generated a pervasive state of anxiety, stress about the babies’ health and themselves, feelings of loneliness, and a fear that the babies would die or forget who they were ^27^ ^,^ ^30^.

#### Synthesized finding 3: challenges to the maternal care of preterm infants imposed by COVID-19

The COVID-19 pandemic and public health measures have impacted maternal proximity to preterm infants and parenting, creating additional challenges for mothers with hospitalized children as well as those at home. Isolation and social distancing have reduced maternal support networks and increased emotional and caregiving burdens as mothers have found themselves alone in motherhood. Similarly, the fragility of premature babies and the possible sequelae meant that the children required more attention at a time when specialized health services were interrupted, damaging health follow-up and leaving the mothers even more helpless. The financial and work situation was also affected by the pandemic, causing more maternal worries and difficulties in providing the necessary care for the children.

This finding was synthesized by aggregating five categories derived from 55 primary findings extracted from the studies ^25^ ^,^ ^26^ ^,^ ^27^ ^,^ ^28^ ^,^ ^29^ ^,^ ^30^ ^,^ ^31^ ^,^ ^34^ ^,^ ^35^ ^,^ ^36^ ^,^ ^38^. Thirty-one of these findings were considered unequivocal, and 24 were credible. The five aggregated categories were: (3.1) specific care for the premature infant, supported by 14 findings (9 unequivocal and 5 credible); (3.2) limitations in parenting with the hospitalized baby, supported by 21 findings (9 unequivocal and 12 credible); (3.3) decreased family support, supported by 11 findings (9 unequivocal and 2 credible); (3.4) increased social vulnerability, supported by 4 findings (2 unequivocal and 2 credible); (3.5) increased maternal burden and stress, supported by 5 findings (2 unequivocal and 3 credible).

### (a) Category 3.1: specific care for the premature infant

Previous knowledge about the development of a full-term baby was not useful in promoting a sense of maternal competence in the face of a premature baby, requiring greater support from the health team to guide mothers in their care ^28^. Premature birth, as an unexpected situation, made it difficult for mothers to prepare for the specific challenges of their children ^28^. They understood that these newborns required special care and, despite recognizing the role of the team, they saw themselves as essential in the care, desiring more opportunities to care and strengthen the bond during their stay in the NICU ^26^ ^,^ ^28^.

Mothers said that hospital discharge preparation should be enhanced and that support with supplies and logistics at home should be increased ^28^ ^,^ ^29^. Many mothers were concerned about the challenges of feeding their premature infants at home ^28^, which they approached by devoting themselves fully to care, compensating for the limitations of the pandemic ^31^. In case of illness, mothers sought medical care promptly, notwithstanding the uncertainty that arose due to the paralysis of some health services during the pandemic ^31^.

### (b) Category 3.2: limitations in parenting with the hospitalized baby

Restrictions on visits to neonatal units have impeded parental care, thereby limiting breastfeeding and skin-to-skin contact ^29^. Conversely, some mothers were reluctant to visit their infants in the NICU due to concerns about contamination ^34^. The use of masks was also perceived as a potential barrier in developing a bond between the mother and infant ^27^. Consequently, numerous mothers felt unprepared and lacked confidence in their maternal abilities ^28^ ^,^ ^29^ ^,^ ^30^. The prolonged separation from their children resulted in a sense of having missed crucial moments with them, which in turn led to a feeling of estrangement and a disruption of the emotional bond with their babies ^29^ ^,^ ^30^.

Mothers viewed the restriction of access to the NICU as an infringement on their rights as parents ^29^. Restricting fathers’ access similarly resulted in delays to the establishment of bonds between parent and child, placing additional burdens on mothers ^25^. As a result, they felt devalued and disenfranchised, which served to exacerbate feelings of isolation ^29^ ^,^ ^38^. Similarly, those who contracted COVID-19 and were required to isolate from their infants experienced a sense of sadness and challenges at the outset of their parenting roles ^30^. Nevertheless, mothers who utilized family surrogates during the hospitalization of their newborns did not face this obstacle ^30^.

Other challenges to parenting during the pandemic included a lack of suitable accommodations for families who had children in hospitals, long distances between homes and hospitals, and the need to work in the fields, as rural families depended financially on agricultural production ^26^ ^,^ ^28^. This meant that many mothers did not learn to recognize their children’s needs and did not adequately prepare for the care of their babies after discharge, making the transition home abrupt or frightening ^28^ ^,^ ^29^.

### (c) Category 3.3: decreased family support

The pandemic profoundly impacted the mothers’ support network, particularly when the family included individuals at heightened risk of infection with SARS-CoV-2 ^28^ ^,^ ^34^ ^,^ ^35^. Frequently, family support was constrained to the baby’s father ^28^. Additionally, the prohibition on paternal access to hospital units excluded them from care and left mothers to guide the fathers about their premature infants ^25^ ^,^ ^27^ ^,^ ^29^. Similarly, the prohibition on family participation in the NICU precluded mothers from sharing moments with their infants and receiving support ^29^. Following discharge, they confronted new challenges due to the lack of expected family support ^28^ ^,^ ^36^. Social isolation measures also made it challenging for them to obtain emotional support from friends during this period ^28^.

### (d) Category 3.4: increased social vulnerability

The prolonged periods of accompanying the infant in the NICU resulted in significant economic distress ^36^. Employment loss and the economic impact of the pandemic have increased social vulnerability and maternal concerns ^31^ ^,^ ^36^. Moreover, the prevalence of domestic violence exacerbated the distress experienced by some mothers, resulting in a sense of helplessness in caring for their children at a time when they could not rely on alternative sources of assistance ^31^.

### (e) Category 3.5: increased maternal burden and stress

The COVID-19 pandemic has been associated with increased maternal burden and stress ^25^ ^,^ ^31^ ^,^ ^36^. Limiting fathers’ visits to neonatal units at times when only mothers had access resulted in a significant burden on mothers in their role. This included difficulties in expressing milk and participating in medical visits ^25^. Following discharge, care required a higher level of patience and dedication on the part of the mother, due to the specific needs of premature children ^31^. Mothers faced challenges in reconciling domestic responsibilities with their children at home, particularly those with older children, due to the closure of schools and leisure facilities ^31^ ^,^ ^36^. This resulted in a more challenging and inflexible routine with limited time for personal activities ^31^.

#### Synthesized finding 4: maternal coping strategies during the pandemic

During the pandemic, mothers have employed various coping strategies to obtain practical support for their children’s care and emotional strengthening. Media reports about the coronavirus have assisted these women in adhering to protective measures, reorganizing their daily lives, and adapting to infant care practices, despite a lack of specific guidelines for premature babies.

This finding was synthesized by aggregating eight categories derived from 41 primary findings extracted from the studies ^25^ ^,^ ^26^ ^,^ ^27^ ^,^ ^28^ ^,^ ^29^ ^,^ ^30^ ^,^ ^31^ ^,^ ^32^ ^,^ ^34^ ^,^ ^35^ ^,^ ^36^. Twenty-nine of them were unequivocal, and 12 were credible. The five aggregated categories were: (4.1) knowledge and opinions about health measures against COVID-19, supported by 8 findings (5 unequivocal and 3 credible); (4.2) adaptation of daily habits and activities, supported by 16 findings (13 unequivocal and 3 credible); (4.3) internal resources for emotional resilience, supported by 4 findings (2 unequivocal and 2 credible); (4.4) resources for social support and connection, supported by 6 findings (4 unequivocal and 2 credible); (4.5) support from health professionals, supported by 7 findings (5 unequivocal and 2 credible).

### (a) Category 4.1: knowledge and opinions about health measures against COVID-19

The mothers demonstrated a high level of awareness about the coronavirus, including its modes of transmission and prevention strategies, largely due to their engagement with the news media ^34^ ^,^ ^36^. Television had a significant role in the dissemination of this information, facilitating the identification of the primary symptoms of the disease and the comprehension of the necessary protective measures ^34^ ^,^ ^36^. However, the specific information regarding the risks posed by COVID-19 to premature babies was notably limited ^27^. Opinions on social isolation were diverse. While there was support for the measure, there were also protests on the difficulties of staying at home for so long ^36^. Nevertheless, some mothers appreciated the perceived positive effects of the pandemic, such as the general social tranquility, the sense of peace, and the opportunity to focus exclusively on the baby without distractions ^25^.

### (b) Category 4.2: adaptation of daily habits and activities

The pandemic has forced mothers to change their daily habits and routines to take preventive measures ^34^. Motivated by the need to protect their children, many have followed safety recommendations such as reducing outings, increasing isolation, frequent hand washing, and wearing masks ^31^ ^,^ ^32^ ^,^ ^34^ ^,^ ^35^. Although television has been an important source of information about the pandemic, some mothers have reduced their exposure to news programs to avoid the emotional overload of too much information ^35^.

Acceptance of restrictions on access to the NICU was greater at the beginning of the pandemic when more restrictive travel policies were implemented, and changes in daily routines to minimize the risk of contagion, such as staying home longer ^25^ ^,^ ^27^ ^,^ ^35^ ^,^ ^36^. Fear of contamination led many mothers to limit their older children’s outings, both to protect them and to avoid transmitting the virus to premature infants ^28^.

To take care of their mental health during the pandemic, mothers broadened their occupational repertoire by engaging in activities such as meditation, reading, listening to music, and watching movies ^35^. They also sought to diversify their childcare strategies to keep children occupied and entertained ^31^.

### (c) Category 4.3: internal resources for emotional resilience

Some mothers resorted to religiosity to maintain a hopeful and strong attitude in the face of the pandemic situation and their children’s hospitalization ^26^ ^,^ ^30^. Pre-pandemic experiences, such as a quiet lifestyle, helped some mothers cope with social isolation ^31^. Additionally, a personal history of prematurity made them more resilient when faced with separation from their infants in the NICU ^30^.

### (d) Category 4.4: resources for social support and connection

Many mothers reported using multi-platform communication apps to try to stay close and introduce their babies to other family members ^28^ ^,^ ^35^. Mothers have found it helpful to share their experiences with other mothers of preterm infants via support and self-help networks ^25^ ^,^ ^28^ ^,^ ^35^. However, pandemic-induced isolation created barriers in forming these connections ^28^. Some of them participated in virtual groups on social networks to ask questions, talk, cry together, and support each other, continuing these interactions months after their babies were discharged ^25^.

### (e) Category 4.5: support from health professionals

Effective communication between health professionals and mothers was essential during the NICU experience, as was the emotional support and validation of their feelings, which helped them cope with the challenges ^26^ ^,^ ^27^ ^,^ ^28^ ^,^ ^29^. Mothers who spent more time in the NICU received more support from the staff and were more involved in their baby’s care ^28^. Support from the healthcare team was also crucial during the transition home ^28^. Additionally, some mothers sought help from professionals they knew before their baby’s birth ^28^.

## Discussion

The vulnerability of preterm infants and the potential sequelae of preterm birth and hospitalization require increased attention from specialized health services. During the pandemic, many of these services were disrupted or functioned inadequately, jeopardizing these children’s care and distressing their mothers. This situation was further exacerbated by the increase in social vulnerabilities resulting from unemployment, financial insecurity, reduced social support, and confinement, threatening the well-being of families ^1^ ^,^ ^12^ ^,^ ^39^ ^,^ ^40^.

Prematurity is a high-risk factor for infant morbidity and mortality and can affect neurodevelopment and have adverse long-term consequences ^41^ ^,^ ^42^. Complications related to prematurity are the leading cause of death in children under five, accounting for 16% of deaths in this group and 35% of neonatal deaths ^43^. Therefore, quality care during hospitalization and follow-up after discharge is essential to mitigate adverse outcomes, with the involvement of the infants’ families, especially the mothers.

However, one of the immediate impacts of the pandemic on families with premature children has been the reduction in access to neonatal units. Although the separation of preterm infants from their caregivers is a problem that predates the pandemic, it has been exacerbated in this context, threatening important care practices such as kangaroo care and exclusive breastfeeding. In many places, restrictions introduced or exacerbated by the pandemic are still in place ^1^.

The results of this review corroborate another study ^44^ that highlighted how policies restricting NICU visitation limited mothers’ interaction with their infants, reducing their ability to participate in care and the time they spent bonding with their babies. These strict policies also impacted the availability of lactation support services, hindering breastfeeding and skin-to-skin contact ^25^ ^,^ ^29^.

In addition, this review found preterm birth and hospitalization in the neonatal unit during the pandemic significantly impacted maternal mental health. The already challenging experience of having a preterm baby was intensified, leading to increased feelings of fear, anxiety, sadness, helplessness, and uncertainty about the future, which affected mothers’ confidence in their parenting skills. These findings are consistent with a quantitative study of women who were pregnant and had children in 2020 ^45^, in which 32.5% of women reported symptoms related to common mental disorders between six months and one year postpartum. Additionally, 62.1% of participants reported a decrease in personal income, and 78.6% reported a decrease in family income due to the pandemic, factors that contribute to psychological distress.

The review also identified that the transition from the NICU to home was particularly stressful and challenging for mothers. The demanding nature of caring for a preterm infant left many mothers feeling unprepared, due to limited contact with their infant during hospitalization. The lack of professional and familial support, exacerbated by pandemic-related movement restrictions, increased the isolation from family and friends, thereby limiting the support available for infant care. Other studies ^44^ ^,^ ^46^ have indicated that some families opted to restrict access to their homes out of fear of infection.

The review found that mothers relied on information from news reports to modify habits and routines to protect themselves and their infants during the pandemic. However, there was a lack of clear information regarding the specific risks COVID-19 posed to preterm infants. These findings align with a scoping review ^12^ on the impact of the pandemic on maternal mental health, early childhood development, and parenting practices, which indicated that restrictive policies heightened mothers’ anxiety, in part because of barriers in accessing clear and sufficient information about the health risks associated with COVID-19.

Given the recurring nature of health crises throughout history, the lessons learned from the COVID-19 pandemic must be utilized to better prepare health services for mothers and preterm infants in future emergencies. Therefore, it is recommended that health services develop humanized, sensitive, and empathetic strategies, recognizing that mothers of hospitalized preterm infants are emotionally fragile and highly value team support. Strengthening counseling about the infant’s health and needs during hospitalization and after discharge is essential to promote dialogue and shared decision-making.

Mothers appreciate having accessible and knowledgeable professionals to advise them about their baby’s health ^47^ ^,^ ^48^, which impacts their satisfaction with care and maternal decision-making. Positive relationships between mothers and healthcare teams enhance parental competence, encourage early breastfeeding, and significantly reduce stress in the NICU ^48^ ^,^ ^49^. Therefore, it is important to foster trust between mothers and professionals to improve mothers’ coping abilities and parenting skills.

Institutions should also encourage support groups among families of preterm infants, including virtual formats, as these groups enhance mutual support and self-care. Additionally, facilities need to be improved to ensure greater accessibility and comfort for mothers during their children’s hospitalization, and environments that enable mothers to remain close to their infants even in isolation situations should be created, such as neonatal units with glass windows. During crises, it is essential to have an adequate number of mental health professionals available to support families. Telecare strategies should be strengthened to maintain a sense of connection when mothers cannot be physically present.

## Conclusions

This study aimed to highlight the experiences of mothers of preterm infants during the COVID-19 pandemic. This review revealed disorganization in the health services and inconsistent rules for visiting neonatal units. Restrictions or interruptions in care for babies and mothers negatively impacted trust in health professionals, mother-child bonding, breastfeeding, and monitoring of premature babies’ development. Despite the implementation of telemedicine services in some facilities, the physical absence of mothers during the care of hospitalized infants or the lack of in-person follow-up of premature infants made them feel alienated or isolated as caregivers responsible for their children.

The strength of this review lies in the inclusion of 14 articles with diverse findings that describe the experiences and challenges that were faced, illustrating how isolation and social distancing measures, although necessary, negatively affected mothers and infants. Furthermore, it produced recommendations based on qualitative evidence to inform public policies that support mothers, provide resources for coping with adverse experiences, and enhance mothers’ skills in caring for preterm infants.

However, there are limitations. Despite efforts to include all relevant studies, it is possible that some were not included in the search. Additionally, the searches revealed a predominance of quantitative studies, which do not necessarily illuminate the nuanced experiences of women with their preterm infants. In turn, most qualitative studies focused on experiences during hospitalization, providing limited information about experiences after discharge.

Further studies using qualitative research methods are needed to gain a deeper understanding of parenting practices after the baby is discharged and the mother-child relationship in the post-pandemic context. Research exploring fathers’ experiences in this context is also essential. Additionally, it is important to investigate families’ perceptions of neonatal services, identifying which care practices were systematically implemented during the pandemic and which ones remain in place.
